# Accurate Diagnosis and Survival Prediction of Bladder Cancer Using Deep Learning on Histological Slides

**DOI:** 10.3390/cancers14235807

**Published:** 2022-11-25

**Authors:** Qingyuan Zheng, Rui Yang, Xinmiao Ni, Song Yang, Lin Xiong, Dandan Yan, Lingli Xia, Jingping Yuan, Jingsong Wang, Panpan Jiao, Jiejun Wu, Yiqun Hao, Jianguo Wang, Liantao Guo, Zhengyu Jiang, Lei Wang, Zhiyuan Chen, Xiuheng Liu

**Affiliations:** 1 Department of Urology, Renmin Hospital of Wuhan University, Wuhan 430060, China; 2 Institute of Urologic Disease, Renmin Hospital of Wuhan University, Wuhan 430060, China; 3 Department of Pathology, Renmin Hospital of Wuhan University, Wuhan 430060, China; 4 Division of Nephrology, Renmin Hospital of Wuhan University, Wuhan 430060, China; 5 Department of Hepatic-Biliary-Pancreatic Surgery, Renmin Hospital of Wuhan University, Wuhan 430060, China; 6 Department of Breast and Thyroid Surgery, Renmin Hospital of Wuhan University, Wuhan 430060, China

**Keywords:** bladder cancer, deep learning, diagnosis, prognostic prediction, whole slide imaging, overall survival

## Abstract

**Simple Summary:**

Early diagnosis and treatment are essential to reduce the mortality rate of bladder cancer. However, current techniques of diagnosis are susceptible to pathologist variability, and histopathological prognostic methods are insufficient to cover all features of muscle-invasive bladder cancer. In this work, we developed weakly supervised models based on deep learning for the diagnosis of bladder cancer and prediction of overall survival in muscle-invasive bladder cancer patients using whole slide digitized histological images in two cohorts. Encouragingly, results showed that our models can not only assist clinicians in the accurate diagnosis of bladder cancer, but also facilitate differential risk stratification in patients with muscle-invasive bladder cancer and improve personalized treatment decisions accordingly. Furthermore, the regions most relevant for diagnosis or prognosis can be further analyzed to increase the amount of information extracted from pathological images. Finally, we identified six genes closely related to cancer progression based on the predicted risk scores, which potentially led to new biomarker discoveries.

**Abstract:**

(1) Background: Early diagnosis and treatment are essential to reduce the mortality rate of bladder cancer (BLCA). We aimed to develop deep learning (DL)-based weakly supervised models for the diagnosis of BLCA and prediction of overall survival (OS) in muscle-invasive bladder cancer (MIBC) patients using whole slide digitized histological images (WSIs). (2) Methods: Diagnostic and prognostic models were developed using 926 WSIs of 412 BLCA patients from The Cancer Genome Atlas cohort. We collected 250 WSIs of 150 BLCA patients from the Renmin Hospital of Wuhan University cohort for external validation of the models. Two DL models were developed: a BLCA diagnostic model (named BlcaMIL) and an MIBC prognostic model (named MibcMLP). (3) Results: The BlcaMIL model identified BLCA with accuracy 0.987 in the external validation set, comparable to that of expert uropathologists and outperforming a junior pathologist. The C-index values for the MibcMLP model on the internal and external validation sets were 0.631 and 0.622, respectively. The risk score predicted by MibcMLP was a strong predictor independent of existing clinical or histopathologic indicators, as demonstrated by univariate Cox (HR = 2.390, *p* < 0.0001) and multivariate Cox (HR = 2.414, *p* < 0.0001) analyses. The interpretability of DL models can help in the analysis of critical regions associated with tumors to enrich the information obtained from WSIs. Furthermore, the expression of six genes (ANAPC7, MAPKAPK5, COX19, LINC01106, AL161431.1 and MYO16-AS1) was significantly associated with MibcMLP-predicted risk scores, revealing possible potential biological correlations. (4) Conclusions: Our study developed DL models for accurately diagnosing BLCA and predicting OS in MIBC patients, which will help promote the precise pathological diagnosis of BLCA and risk stratification of MIBC to improve clinical treatment decisions.

## 1. Introduction

Bladder cancer (BLCA) is one of the most common tumors worldwide, with approximately 573,000 new cases and 213,000 deaths in 2020 [1]. According to the IDENTIFY study, the largest international prospective study of patients with suspected urinary tract cancer, BLCA is the most prevalent cancer diagnosis in patients with hematuria [2]. The five-year survival rate for patients with non-muscle-invasive bladder cancer (NMIBC) is estimated to be around 90%, but the five-year survival rate for patients with muscle-invasive bladder cancer (MIBC) decreases dramatically as the tumor invades different layers of the bladder [3]. Recently, the IDENTIFY study presents a multivariable prediction model for the detection of urinary tract cancers, which assists clinicians in early risk assessment, but only in patients with hematuria [4]. Despite the advances in surgery and other diagnosis and treatment techniques over the past 30 years, clinical outcomes of BLCA have not substantially improved [5]. Given that early detection, accurate diagnosis, and appropriate therapeutic intervention are critical for reducing the mortality of BLCA, precise and consistently effective methods of pathologic assessment are essential.

Currently, the diagnosis of BLCA is carried out by pathologists through biopsy, which serves as the gold standard. This typically requires pathologists to manually review each pathological slide and rely on personal expertise to make an accurate diagnosis. Such manual analysis is not only time-consuming, labor-intensive, and tedious, but is also subject to observer variability. Moreover, the shortage of expert pathologists has become a global problem, which, to a certain extent, causes an increase in the workload of available pathologists [6]. Hence, it is necessary to develop a convenient, efficient, and accurate method to diagnose BLCA using pathological slide images.

The pathological type of BLCA can be low-grade or high-grade; high-grade BLCA should be treated more aggressively and is more likely to result in death [7]. It turns out that in most human cancers, including BLCA, prognosis is closely related to pathological criteria [8]. Histopathological classification through the tumor-node-metastasis (TNM) staging system jointly developed and established by the American Joint Committee on Cancer (AJCC) and the Union International Committee on Cancer has some prognostic and therapeutic value, but is insufficient to cover all clinical characteristics of BLCA patients and the heterogeneity of patient outcomes [9]. Furthermore, risk stratification relying on histopathological staging is susceptible to variability in observation and judgment among pathologists. Accordingly, there is an urgent need to develop robust and reproducible methods to identify predictive markers consistently associated with survival in MIBC patients.

In recent years, the use of artificial intelligence has been greatly beneficial in pathology and has tremendously facilitated the growth of digital pathology. The advent of deep learning (DL) and the availability of thousands of digitized whole slide images (WSIs) may provide new opportunities for the diagnosis and prediction of disease outcomes [10]. DL can adaptively extract relevant image features according to learning objectives for tasks such as classification, segmentation, and detection [11,12,13]. It has been reported that an algorithm based on DL can identify bladder tumors with a specificity of up to 98.6% [14]. A previous study [15] used DL to successfully predict the molecular subtypes of MIBC by processing hematoxylin and eosin (H&E) slides with a similar or superior performance compared to that of pathologists.

There is growing evidence [16,17,18,19] indicating that automated analysis of WSIs can improve disease diagnosis and prognosis prediction, thereby enhancing treatment options and maximizing efficacy. To solve the time-consuming and laborious problem of manual annotation, unsupervised or weakly supervised DL models are gaining popularity. Courtiol et al. [20,21] used DL to develop models that could accurately predict patient survival in hepatocellular carcinoma and mesothelioma, respectively, without the need for local annotated regions provided by any pathologist. Lu et al. [22] reported an interpretable weakly supervised DL method for binary and multi-class WSI classification using only slide-level labels without any additional manual annotations. Furthermore, DL has been shown to predict the expression of differential genes or molecular biomarkers from pathological images of BLCA, such as PD-L1 [23] and FGFR [24,25], which is cheaper, more effective, and more robust than next-generation sequencing or immunohistochemical staining methods. Therefore, a possible solution is to utilize DL to extract potential clinical and/or biological information in WSIs for diagnosis and prediction of overall survival (OS) in MIBC patients.

In this study, we developed a DL-based diagnostic model for BLCA patients and a prognostic model for MIBC patients, named BlcaMIL and MibcMLP, respectively, and demonstrated their effectiveness in tumor diagnosis and prognosis prediction on two cohorts. The results showed that BlcaMIL can accurately distinguish between tumor and normal tissues, and MibcMLP is more accurate than the use of most clinical information and pathological features in predicting the OS in MIBC patients. By visualizing the region of interest (ROI), it is possible to gain insights into the most relevant features of DL-models for diagnosing and predicting patient outcomes.

## 2. Materials and Methods

### 2.1. Patient Cohorts

We retrospectively analyzed two cohorts of BLCA patients for this study. The first cohort was from The Cancer Genome Atlas (TCGA) public dataset consisting of a total of 926 H&E-stained WSIs from 412 BLCA patients, of which 887 were tumor and 39 were normal. Given that the uneven distribution of tumor and normal images in TCGA cohort, the data augmentation technique was used to address this imbalance issue. An independent external dataset was obtained from the Renmin Hospital of Wuhan University (RHWU; Wuhan, Hubei, China) comprising 250 H&E-stained WSIs obtained from 150 BLCA patients from 2017 to 2022, of which 150 were tumor and 100 were normal.

For the development of diagnostic model, the inclusion criteria for both cohorts were (a) specific pathological diagnosis of BLCA and (b) availability of clear H&E-stained pathological slides.

The TCGA cohort provides two types of H&E-stained WSIs: tissue slides and diagnostic slides. Tissue slides are sections of frozen tumor specimens that are often used to determine whether tumor boundaries are clean. Diagnostic slides are formalin-fixed paraffin-embedded sections, which generally preserve cell morphology better and are more situable for computational analysis.

We adopted the following inclusion criteria for developing the prognostic model once the criteria for the diagnostic model were met: (a) availability of clinicopathological information, (b) availability of follow-up data, (c) specific pathological diagnosis of MIBC and (d) use of diagnostic slides rather than tissue slides.

In addition, we collected the corresponding clinical data as well as biological and pathological characteristics of the patients (including age, gender, lymphovascular invasion, tumor size, OS, survival status, pathological grade and TNM staging (according to AJCC 8th Edition Staging Manual [26]) for survival analysis of the prognostic model. The patient data for the TCGA cohort were collected through the UCSC Xena database (https://xenabrowser.net/datapages/, accessed on 22 October 2022), and patient data for the RHWU cohort were obtained through the hospital information management system.

### 2.2. WSI Preprocessing

The WSIs from the two cohorts had different magnifications. Specifically, WSIs from the TCGA cohort had an original magnification of 40× (without fixed size, the image size could be larger than 100,000 × 100,000 pixels), whereas those from the RHWU cohort had an original magnification of 20× (64,000 × 58,000 pixels). Therefore, we uniformly processed these WSIs to 20× magnification and used the resulting WSIs in the next step.

Since the WSIs contained extremely rich content (up to a resolution of 100,000 × 100,000 pixels) and could not be directly used for model training, they were pre-processed first. We loaded WSIs at 10× magnification (0.5 μm/pixel), segmented out tissue regions using an area threshold filtering-based approach, and then used the openslide-python toolkit (https://openslide.org/, accessed on 22 October 2022) to divide WSIs into small images of the same fixed-out size (448 × 448 pixels), each of which is called a patch. Each patch had to contain 80% of the tissues to be included, and had the same label as the pathological diagnosis of the WSI itself. A color threshold was used to exclude possible background images from patches. Due to the heterogeneity in staining protocols used to obtain the WSIs by different centers, we unified the colors of all patches using the structure-preserving color normalization method proposed by Vahadane [27] and Anand [28].

### 2.3. Feature Extraction and Reduction

We used Resnet-50 to extract 2048 relevant features for each patch. The network was pre-trained on the ImageNet dataset (over one million images) and had been shown to accurately identify over a thousand categories [29]. At that point, we had constructed a 2000 × 2048 vector for each WSI. Because the data dimensions were too high and prone to overfitting during model training, we used the trained autoencoder for dimensionality reduction. This autoencoder included a hidden layer of 512 neurons, which reduced the input dimension of the prediction part from 2048 to 512 to avoid potential problems of overfitting, long processing time, and heavy usage of computational memory. We randomly selected 200 patches (66,000 patches in total) from each WSI to train the autoencoder, and the MSE loss was reduced to 0.0052 after 100 epochs.

### 2.4. Development of Diagnostic Model

BlcaMIL is an end-to-end weakly supervised neural network, which combines the attention strategy with multiple instance learning (MIL) algorithm, and can be used for the task of binary classification of the entire WSI. The theoretical basis of the MIL algorithm is that, assuming a WSI is a bag, then all patches it contained are instances of the bag. When a WSI is marked positive, at least one patch is positive; when a WSI is marked negative, then all patches are negative. Based on the assumptions of the MIL algorithm, which had been widely used for weakly supervised positive/negative binary classification, we added an additional attention mechanism to it.

During training and inference, the attention network in the BlcaMIL assigned an attention score to each patch, representing its importance to the overall WSI classification. We input the extracted patch-level features into the BlcaMIL, and aggregated them into a WSI-level representation through an average pooling function, which was used to predict the probability score for the final diagnosis.

### 2.5. Development of Prognostic Model

MibcMLP is also a weakly supervised neural network consisting of a two-step algorithm. To generate a risk score for each patch, we used a one-dimensional dense layer. The dense layer was composed of 512 neurons. The 512 features extracted from each patch were weighted and summed (the weights were obtained after model learning), and the risk score was calculated. Subsequently, we sorted the risk scores of the 2000 patches, selected the 25 highest and 25 lowest scoring patches, formed a vector of size 50, and used it as the input for the final step. This operation allowed us to clearly understand which patches were finally used as input for the risk prediction step, thereby facilitating subsequent interpretation of the ROIs for the DL-model.

The final step was a multilayer perceptron consisting of two fully connected layers with 200 and 100 neurons, each with a sigmoid activation function. This was a critical step in predicting the prognosis of MIBC patients, and its function was to convert the 50-patch risk scores into a survival-related risk score that was representative of the entire WSI.

**Figure 1:**
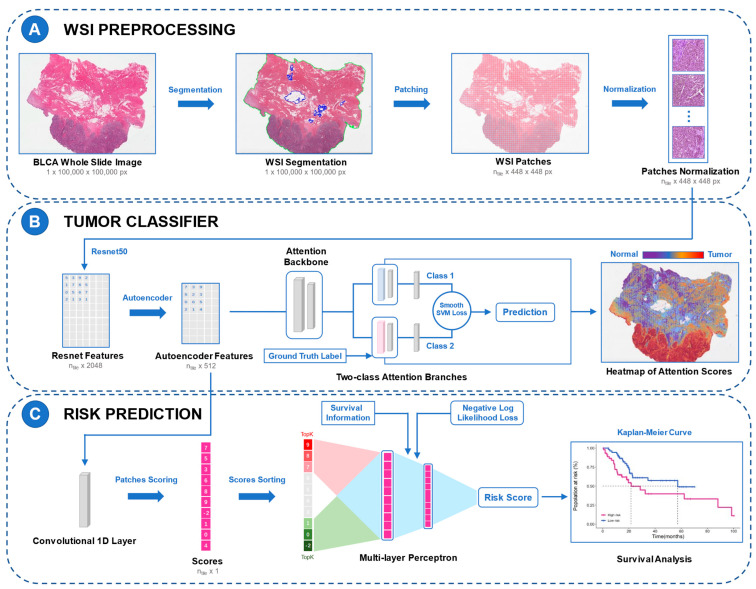
Study flow chart and the layouts of the DL models. The framework of BlcaMIL is shown in A and B and the framework of MibcMLP is shown in A and C. (**A**) Each WSI was first segmented into tissue-containing regions (green border) and empty regions inside the tissue (blue border), and then patches with 448 × 448 pixels were generated. (**B**) Feature extraction was performed on all patches using ResNet-50, and dimensionality reduction was performed with Autoencoder. Through the MIL model with attention mechanism, the extracted patch-level features were input into the BlcaMIL model, the attention scores of these patches were output, and the average pooling function was used to aggregate them into the WSI-level to make the final diagnosis. Heatmaps visualize ROIs for the model. (**C**) Patch-level features were fed into the network along with survival information, and each patch was assigned a risk score through an iterative learning process. Then, the 50 patches with the highest and lowest scores were selected to be input to the MLP model to predict patient survival. Finally, MIBC patients were stratified using the resulting risk scores. DL, deep learning; WSI, whole slide image; MIL, multiple instance learning; ROI, region of interest; MLP, multi-layer perceptron; MIBC, muscle invasive bladder cancer.

The loss functions for the diagnostic and prognostic models used smooth top1 SVM loss and the negative log-likelihood function of the Cox proportional hazards model, respectively. The training set was repeatedly validated using a five-fold cross-validation strategy, using internal and external validation. The layouts of the two DL models are shown in Figure 1.

### 2.6. Model Interpretability and Visualization

For the interpretability of the diagnostic model, the attention scores of BlcaMIL-predicted categories were converted to percentiles and values between 0 and 1 (1 for the most contribution, 0 for the least). The normalized scores were then converted to RGB colors using a divergence colormap, mapped to their corresponding spatial locations in the WSI to generate an attention heatmap. The color red represented areas to which the neural network paid close attention, while the color blue represented areas receiving less attention. Furthermore, the BlcaMIL also indicated some patches with high attention scores, which was convenient for review, and helped in understanding the ROIs of the DL-model and explaining the tumor-related pathological features.

The interpretability of the prognostic model was high because we knew exactly which patches were used to create the risk scores. We extracted the scores of all patches, selected the 100 patches with the highest and 100 with the lowest scores after tiling and sorting, and invited two expert uropathologists for the analysis of tumor-related pathological characteristics. The uropathologists did not know the risk scores assigned to these patches in advance, and then made statistical records of tumor-related pathological characteristics for these 200 patches.

### 2.7. Statistical Analysis

The classification performance of the diagnostic model was assessed by the area under the receiver-operator curve (AUC), as well as the accuracy, sensitivity, and specificity. A two-sided McNemar’s test was performed to compare the differences in accuracy, sensitivity, and specificity between the diagnostic model and the pathologists. Cohen’s kappa coefficient was calculated to assess the diagnostic agreement between the diagnostic model and the pathologists. We used Harrell’s concordance index (C-index) as an indicator to evaluate the performance of the prognostic model in predicting OS. The Kaplan–Meier survival curve was plotted using R software (Version 3.5.1, R Core Team, Vienna, Austria) to evaluate the correlation between the risk scores generated by the prognostic model and the OS of MIBC patients, and the Log-Rank test was performed. The R software package was obtained from CRAN (https://cran.r-project.org, accessed on 22 October 2022). Pearson’s correlation test was performed to assess the significance of the correlation between the two covariates. Differences with p values lower than 0.05 were considered statistically significant (two-tailed). Python (Version 3.8.13, CreateSpace, Scotts Valley, CA, USA) and Pytroch (Version 1.10.0, Curran Associates, Inc., Vancouver, BC, Canada) were used for model building and development.

## 3. Results

### 3.1. Patient Characteristics

After screening for the inclusion criteria of the diagnostic model, we included 412 BLCA patients from the TCGA cohort and 150 BLCA patients from the RHWU cohort. A total of 926 WSIs from the TCGA cohort were obtained for training the BlcaMIL model. Through data augmentation, 1627 WSIs (tumor: normal = 887:740) were finally used for the development of the BlcaMIL model. Of those, 80% (*N* = 1302) were randomly selected as the training set while the remaining 20% (*N* = 325) were used as the internal validation set. 250 WSIs from the RHWU cohort were used for independent external validation. The detailed data distribution is shown in Supplementary Table S1.

From the TCGA cohort, 326 patients were eligible according to the inclusion criteria for the prognostic model to participate in the construction of MibcMLP. The TCGA cohort comprised 326 WSIs, which were randomly assigned to the training set (*N* = 190) and internal validation set (*N* = 136). The external validation set included 144 WSIs from the RHWU cohort. Table 1 exhibits the baseline characteristics of the two cohorts used for the prognostic model.

### 3.2. Performance of the Diagnostic Model

A previous study has shown that the discriminative ability of the MIL model for images was optimal at 10× magnification compared to other magnifications [17]. Accordingly, we loaded all WSIs at 10× magnification and extracted a total of 13,115,687 patches (448 × 448 pixels). The labels of these patches were consistent with the corresponding WSI labels. Relevant features were extracted for each WSI using Resnet-50, a pre-trained convolutional neural network based on ImageNet, before training the model.

In the diagnostic model, the accuracy of the training set and that of the internal validation set were both 0.998 (AUC, 1.000). Even in the external validation set, the generalization ability of BlcaMIL was still strong with an accuracy of 0.987 (AUC, 0.993) (Table 2a). In addition, we invited two expert uropathologists A and B, who were chief or associate chief uropathologists, and a junior pathologist C who was under training to judge 250 WSIs from the external validation set. Our diagnostic model BlcaMIL was better than that of the junior pathologist C (Accuracy = 0.876) (*p* < 0.0001, paired Chi-squared test). There was no significant difference when using BlcaMIL compared to expert uropathologist A (Accuracy = 0.991) (*p* > 0.05, paired Chi-squared test) or expert uropathologist B (Accuracy = 0.993) (*p* > 0.05, paired Chi-squared test). Moreover, BlcaMIL achieved decent inter-observer agreement with the expert uropathologists (kappa = 0.909 and 0.925, respectively) (Table 2b).

### 3.3. Performance of the Prognostic Model

We used the C-index metric to assess the ability of the model to predict OS. We found that the MibcMLP model performed well on both the training set and the internal validation set, with C-index values of 0.744 and 0.631, respectively. Based on the input WSI, the MibcMLP model we trained was able to assign each patient a risk score. In contrast to the classification of histopathology, the score was a continuous numerical value rather than a discrete classification. We divided MIBC patients into high-risk and low-risk score groups using the median risk score in the training set as a cut-off point. We then adopted Kaplan-Meier plots and univariate and multivariate Cox models to assess the association between the risk scores and survival outcomes in MIBC patients. In the training set, the risk score predicted by MibcMLP was a strong predictor of OS in univariate analysis (HR = 3.896, *p* < 0.001, Cox analysis; Supplementary Table S2, Supplementary Figure S1). After retaining significant prognostic indicators in univariate analysis (pT stage, pN stage, pM stage, pTNM stage, and Lymphovascular invasion), the risk score remained strongly predictive in multivariate analysis (HR = 3.557, *p* < 0.001, Cox analysis; Supplementary Table S2).

**Figure 2:**
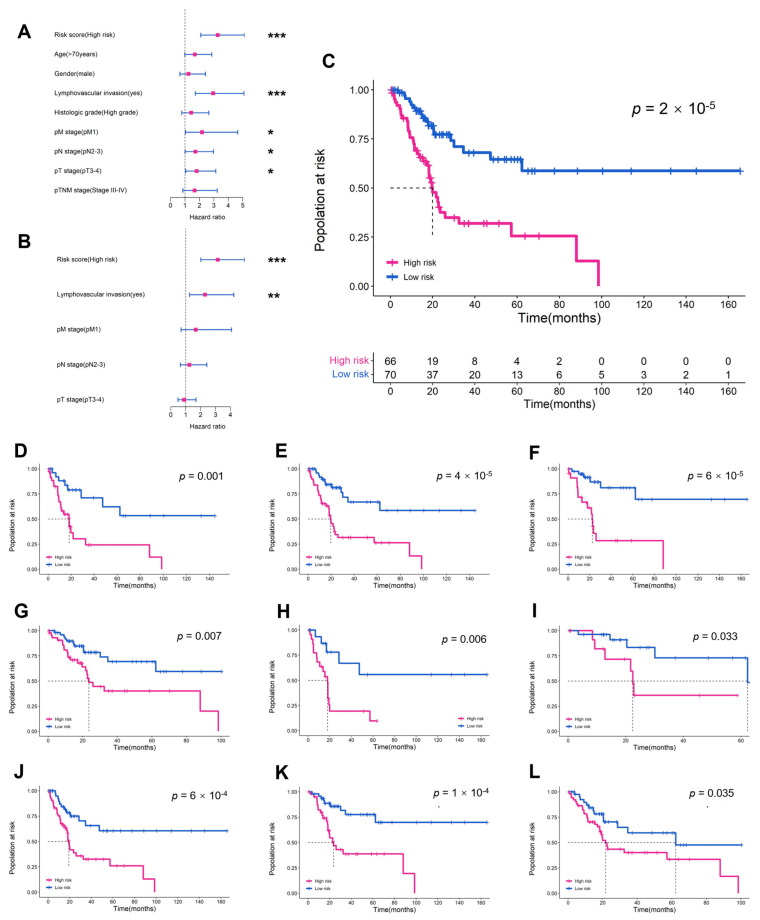
Prognostic value of MibcMLP-generated risk scores in the internal validation set. HR and 95% CI for MibcMLP and other clinicopathological features to predict survival in (**A**) univariate Cox and (**B**) multivariate Cox analyses. MibcMLP model scores were converted to binary scores (high risk or low risk) using the median risk score of the training set as a cut-off. K-M survival curves for (**C**) the entire internal validation set and the following subgroups: (**D**) age ≥70; (**E**) male; (**F**) pT stage 3–4; (**G**) pN stage 0–1; (**H**) pN stage 2–3; (**I**) pTNM stage 1–2; (**J**) pTNM stage 3–4; (**K**) no lymphovascular invasion; (**L**) high histologic grade. ***, *p* < 0.001; **, *p* < 0.01; *, *p* < 0.05; HR, hazard ratio; CI, confidence interval.

In the internal validation set, the MibcMLP model stratified the MIBC population more accurately than any other clinical or pathological variable in Cox univariate analysis (HR = 3.274, *p* < 0.001, Figure 2A). Even after adjusting for significant prognostic indicators in univariate analysis (pT stage, pN stage, pM stage, and Lymphovascular invasion), the risk score predicted survival outcomes in the multivariate analysis (HR = 3.157, *p* < 0.001, Cox analysis; Figure 2C). The risk score predicted by MibcMLP had independent prognostic value (*p* = 2 × 10^−5^, Log-rank test; Figure 2B), even between among other subgroups (such as age, gender, pT stage, pN stage, pM stage, pTNM stage, histological grade and lymphovascular invasion; Figure 2D–L). These results indicated that the pathological features captured by MibcMLP were not redundant with the existing clinicopathological features, and constituted an effective prognostic method independent of current AJCC TNM staging.

**Figure 3:**
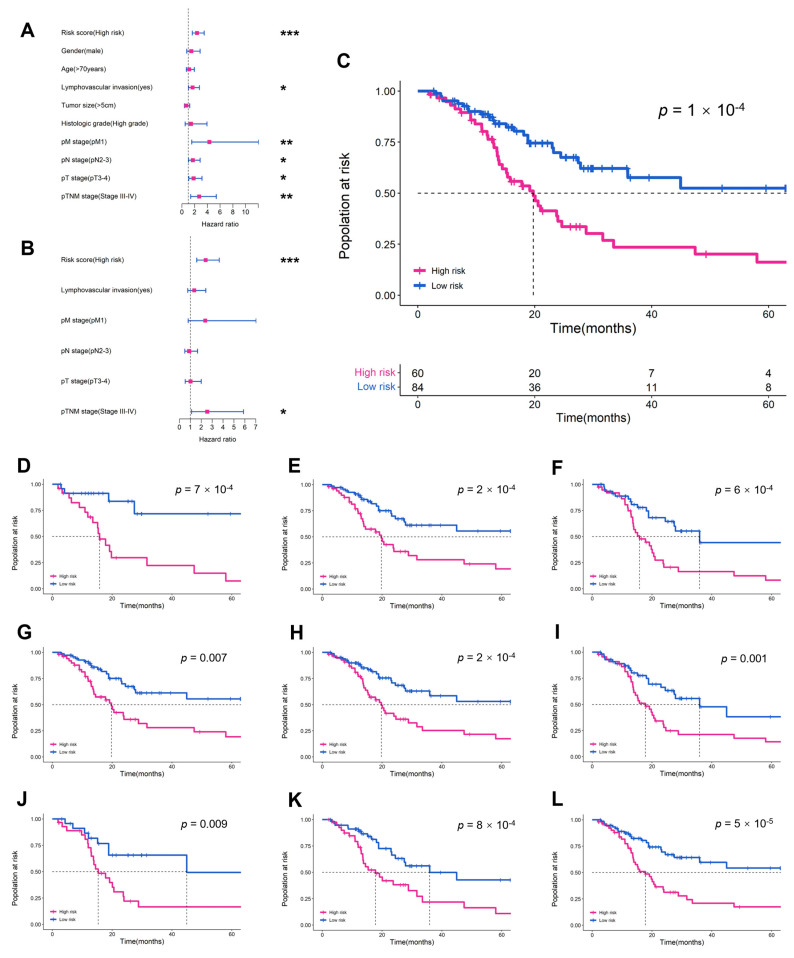
The performance of MibcMLP in predicting prognosis in the external validation set. (**A**) univariate Cox and (**B**) multivariate Cox analyses are exhibited. Using the median risk score in the training set as the cut-off point, (**C**) K-M survival curves for the entire external validation set and the following subgroups: (**D**) age ≥70; (**E**) male; (**F**) pT stage 3–4; (**G**) pN stage 0–1; (**H**) pM stage 0; (**I**) pTNM stage 3–4; (**J**) lymphovascular invasion; (**K**) tumor size <5cm; (**L**) high histologic grade. ***, *p* < 0.001; **, *p* < 0.01; *, *p* < 0.05.

We assessed the robustness of the MibcMLP model by testing it on an independent RHWU cohort. A total of 144 BLCA patients met the inclusion criteria (Table 1). Of these, 65 patients died, and the OS was lower than that in the TCGA cohort (median 16.0 vs. 18.1, *p* = 0.330, Kruskal-Wallis nonparametric test). The following variables were collected and included in subsequent analyses: age, gender, tumor size, pT stage, pN stage, pM stage, pTNM stage, lymphovascular invasion and histological grade. The MibcMLP model extracted and processed patches of 144 WSIs corresponding to 144 patients with a predicted survival C-index of 0.622. We divided the RHWU cohort patients into subclass of high-risk and low-risk using stratification cut-off point from the training set. The results showed that the risk score predicted by MibcMLP was significantly better than other clinicopathological features in the Cox univariate analysis (HR = 2.390, *p* < 0.001, Cox analysis; Figure 3A) and was an independent prognostic factor (*p* = 1 × 10^−4^ Log-rank test; Figure 3B). Multivariate Cox regression analysis showed that MibcMLP was also an independent prognostic variable (HR = 2.414, *p* < 0.001, Cox analysis; Figure 3C) after adjustment for important prognostic indicators. MibcMLP predicted survival well even after stratification for other common clinicopathological features (such as age, gender, pT stage, pN stage, pM stage, pTNM stage, histological grade, tumor size and lymphovascular invasion; Figure 3D–L).

### 3.4. Visualization of DL Models

**Figure 4:**
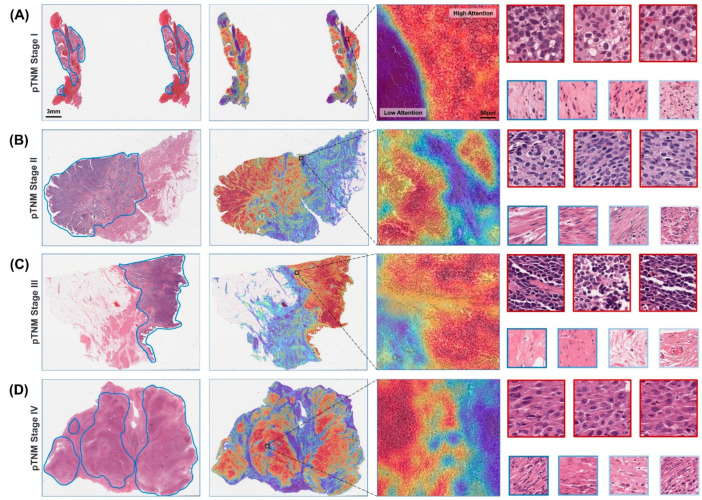
Heatmaps of the diagnostic model on WSIs at different pathological stages. A representative WSI for each pathological stage was annotated by a uropathologist who roughly delineated the tumor tissue area (first column), including (**A**) AJCC TNM stage I, (**B**) stage II, (**C**) stage III and (**D**) stage IV. The attention scores of the predicted categories of patches are calculated by the model, and the attention heatmap corresponding to each WSI was generated and overlaid onto it (second column). It is then further zoomed in to show the heatmap of the ROI, highlighting the tumor and normal borders (third column). Patches with the highest attention (red border) often exhibit well-known tumor morphology, while patches of low interest (blue border) tend to be normal tissue or background (fourth column).

The BlcaMIL model assigned an attention score to each patch, which represents the degree of contribution to the prediction result. We converted the attention scores into heatmaps to visualize the ROIs of the model. As shown in Figure 4, the high-attention areas mostly consisted of tumor cells with a dense arrangement, hyperchromatic nuclei, and high atypia, while the low-attention areas comprised mostly normal tissue or background. This demonstrated a high degree of concordance with annotations of the pathologists regardless of the pathological stage of BLCA, which was consistent with the established experience of detecting tumor regions, in line with human pathology expertise. This simple and intuitive interpretability and visualization technique allowed us to gain insight into the morphological patterns predicted by the model.

**Figure 5:**
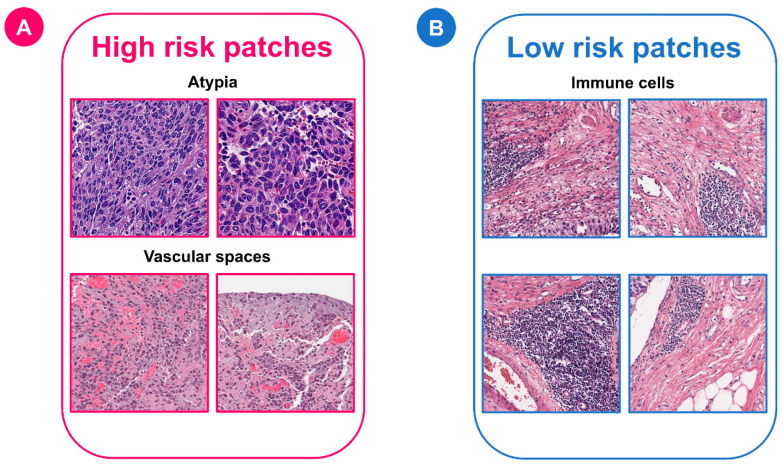
Representative examples of patches classified as high or low risk by the MibcMLP model. The top 200 most predictive patches were analyzed by expert uropathologists who was unaware of the risk scores. (**A**) Features predicting a high mortality risk included cellular atypia and vascular space. (**B**) Features predicting a low risk of death was the presence of immune cells.

For the MibcMLP model, we aggregated all patches together, obtained the risk score of each patch through MibcMLP, picked out the patches with the highest scores (*n* = 100) and the lowest scores (*n* = 100), and tried to interpret their histopathological features. The patches were independently reviewed by two expert uropathologists who were unaware of the scores assigned, and the associated pathological features were recorded. The results showed that most of the patches associated with poor survival were mainly located in the stromal region, not within the tumor region (ratio, low [resp. high] survival patches in stroma = 94/100 (resp. 10/100, Chi-squared test, *p* = 1.4 × 10^−32^). Among the pathological features recorded, the features most predictive of high risk were the presence of vascular space (Chi-squared test, *p* = 3.1 × 10^−20^), high cytological atypia (Chi-squared test, *p* = 4.8 × 10^−34^), and high nuclear pigmentation (Chi-squared test, *p* = 2.7 × 10^−33^). The feature most predictive of low risk was immune cell infiltration (Chi-squared test, *p* = 2.0 × 10^−17^) (Figure 5). Taken together, the above results demonstrated that the BlcaMIL and MibcMLP models can detect histopathological features related to diagnosis and survival in BLCA.

### 3.5. Gene Expression Correlation with Risk Scores

**Figure 6:**
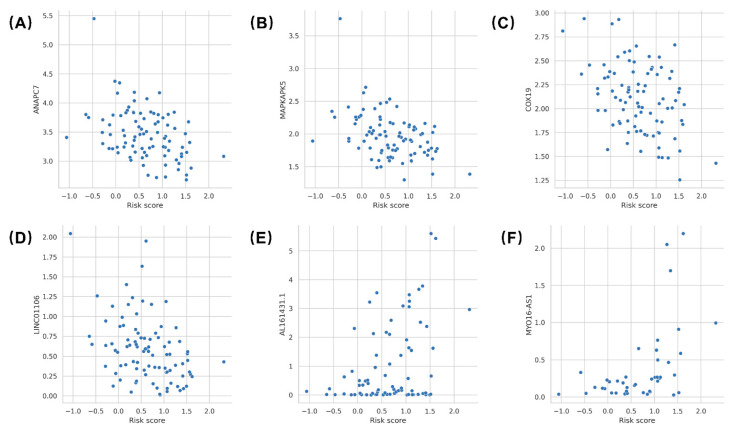
Correlates between risk scores and gene expression levels. Biological correlation between MibcMLP risk scores and (**A**) the ANAPC7 expression (*N* = 90 samples), (**B**) the MAPKAPK5 expression (*N* = 90 samples), (**C**) the COX19 expression (*N* = 90 samples), (**D**) the LINC01106 expression (*N* = 90 samples), (**E**) the Al161431.1 expression (*N* = 78 samples), and (**F**) the MYO16-AS1 expression (*N* = 43 samples) available for the TCGA dataset.

We obtained gene expression data of TCGA MIBC patients from the UCSC Xena database, with up to 56,536 genes. The association between risk scores and gene expression levels in each patient was examined by Pearson correlation analysis, revealing possible potential biological correlations. The expression of six genes was significantly associated with MibcMLP-predicted risk scores: ANAPC7 (correlation = −0.407; Pearson’s correlation test, *p* = 6.9 × 10^−5^), MAPKAPK5 (correlation = −0.443; Pearson’s correlation test, *p* = 1.2 × 10^−5^), COX19 (correlation = −0.412; Pearson’s correlation test, *p* = 5.4 × 10^−5^), LINC01106 (correlation = −0.410; Pearson’s correlation test, *p* = 6.2 × 10^−5^), AL161431.1 (correlation = 0.393; Pearson correlation test, *p* = 3.4 × 10^−4^) and MYO16-AS1 (correlation = 0.497; Pearson correlation test, *p* = 7.1 × 10^−4^) (Figure 6).

## 4. Discussion

BLCA is a disease with complex molecular features, severe morbidity, and high mortality. Mining of potentially robust clinical and/or biological features will aid in the diagnosis and risk stratification of BLCA patients to facilitate personalized treatment. A recent series of studies has explored the impact of immunohistochemical assays [30], conventional serum and histological biomarkers [31,32,33,34,35], and adjuvant therapy [36,37] on longitudinal monitoring and prognosis definition in BLCA patients. In this study, we utilized DL to develop a diagnostic model, BlcaMIL, and a prognostic model, MibcMLP, using WSIs for accurate diagnosis of BLCA and prognosis prediction of MIBC patients. Encouragingly, our BlcaMIL accurately differentiated BLCA from normal pathological images (AUC close to 1), with a performance comparable to that of expert uropathologists and better than that of a junior pathologist. Our MibcMLP not only had excellent performance in the training set (C-index = 0.744) and internal validation set (C-index = 0.631), but also exhibited robust performance on the independent external validation set (C-index = 0.622). Furthermore, univariate and multivariate Cox analyses demonstrated that the risk score predicted by MibcMLP was an independent prognostic factor, a beneficial complement to existing markers, and more accurate than classical clinical, biological, and pathological features in predicting OS.

In recent years, data-driven machine learning and DL have been widely used in the processing and analysis of medical images, providing new tools for disease diagnosis and prognosis prediction. A novel approach combining radiomics and machine learning has brought encouraging results in the diagnosis and prediction of urological cancers [38,39,40]. Previously, our team developed a DL-model based on cystoscopy for clinical real-time recognition of bladder tumors with an accuracy comparable to that of experienced clinical experts [41]. In this study, we adopted DL to analyze digitized H&E-stained BLCA pathological images. There have been some DL studies based on WSIs where the diagnosis and survival prognosis of tumor patients have been successfully analyzed in soft tissue sarcoma [42], brain glioma [43], gastric cancer [17], and rectal cancer [19]. This type of research has been emerging also in the field of BLCA. Wetteland et al. [44] proposed a DL pipeline that identified diagnostic-relevant regions in WSI and predicted the grade. Fuster et al. [45] analyzed pathological images of NMIBC patients and proposed a multi-scale DL model that detected cancerous areas patterns across WSIs for accurate T1 staging. Woerl et al. [15] successfully identified molecular subtypes of MIBC patients based on convolutional neural networks, and visualized the consequent ROIs. Lucas et al. [46] performed an analysis of relapse-prone NMIBC and confirmed that using DL in conjunction with WSIs and clinical data could improve relapse prediction in BLCA patients. However, these recent studies did not attempt to make an accurate diagnosis using the WSIs, nor did they directly analyze the association between WSIs and survival outcomes in MIBC patients.

An increasing number of studies have used BLCA gene expression profiling or genetic testing approaches to predict OS in BLCA patients [47,48,49,50]. Next-generation sequencing approaches can provide the wealth of information required to molecularly classify BLCA patients, thereby identifying potential therapeutic targets [51]. Recently, Lindskrog et al. [52] conducted a comprehensive multi-omics analysis of 834 NMIBC patients from the UROMOL project. Their results demonstrated the independent prognostic value of transcriptomic subtyping and chromosomal instability levels over clinicopathological features and were confirmed in 1228 validation samples. However, implementation of these high-throughput gene expression profiling/RNA-sequencing technologies in clinical practice is currently hindered by high costs, the requirement of nucleic acid extraction, and issues of standardization and reproducibility. In contrast, our diagnostic and prognostic models require only H&E-stained digitized slide images as inputs to make a diagnosis or output a risk score associated with survival. Such slides are readily obtained in a surgical treatment setting due to the abundance of histological material available during surgery. Moreover, the collected WSIs do not require professional pathologists for the annotation of ROIs, as the trained model can automatically identify specific regions associated with tumors in WSIs, greatly reducing the need for pathologists, as well as lowering the time and effort to annotate ahead of time. Furthermore, WSIs contain a wealth of potential information that is often difficult to detect by pathologists. Traditional histological stage and pathological grade prognostic methods are likely to include inter-observer variation, whereas the DL-based method reduces human intervention and potentially improves reproducibility. Consequently, we believe that our methods are more helpful for the diagnosis and risk prediction of BLCA patients in economically underdeveloped areas with a shortage of pathologists.

Although DL tools have produced extremely reliable results so far, the inference process of these models is often highly opaque, making it difficult or impossible for us, and even for a domain expert, to understand. This so-called “black box” model undermines the credibility of the results and limits the practical application of DL in pathology [53]. In our models, we extracted the patches that were identified as the most relevant to diagnosis or prognosis for pathological interpretation. This transparent and interpretable process allowed us to further submit these patches to expert uropathologists for review and analysis. Our results indicated that histopathological features obtained by DL-models, such as high cytological atypia and high nuclear pigmentation, are currently used features for diagnosis and prognosis by pathologists. Moreover, the presence of vascular space is strongly associated with high-risk patients with low OS, which is consistent with the current findings [54]. For low-risk patches, immune cell infiltration is an extremely important feature. The tumor microenvironment plays a crucial role in tumor progression, and massive immune cell infiltration is often associated with molecular subtypes with better prognosis, not only in BLCA but also in other cancers [55,56,57]. Therefore, we believe that the novel features extracted by the DL-model not only enable reliable prognostic analysis of MIBC patients, but also make essential contributions to dissect tumor molecular subtypes.

We also further investigated the association between risk scores and gene expression levels in MIBC patients, in which ANAPC7, MAPKAPK5, COX19, and LINC01106 were negatively correlated with predicted risk scores, and AL161431.1 and MYO16-AS1 were positively correlated with risk scores. Previous studies have shown that these six genes play important roles in promoting or inhibiting the occurrence and development of tumors. ANAPC7 has been identified as a sponge of miR-373 that inhibits tumor growth in vitro and in vivo by regulating the cell cycle [58]. MAPKAPK5 upregulation is associated with high expression of the transcriptional regulator YAP and poor prognosis in clinical tumor samples, and its positive regulation of YAP activity plays an important role in human cancers [59]. COX19 may affect the assembly of cytochrome C oxidase (COX) subunits, which in turn affects the activity of COX and apoptosis [60]. COX19 has been confirmed to be a key factor in the inhibition of tumor cell apoptosis by microRNA-21, and inhibition of COX19 expression may enhance apoptosis and reduce tumor cell proliferation [61]. LINC01106 has been shown to promote the progression of BLCA and its expression is enhanced in BLCA. Knockdown of LINC01106 results in the inhibition of proliferation, migration, and invasion of BLCA cells, making LINC01106 a potential target for the treatment of BLCA patients [62]. AL161431.1 is a long non-coding RNA associated with tumor hypoxia and autophagy [63,64]. It exhibits an elevated level in pancreatic cancer [65], endometrial cancer [66] and lung cancer [67], and may promote the migration and invasion of tumor cells. MYO16-AS1 is a strong prognostic factor in MIBC, and its upregulation is associated with longer OS in MIBC patients, suggesting that it plays a cancer-promoting role in MIBC [68]. Therefore, some genes closely related to the progression of MIBC can be identified through the predicted risk score, which can provide a reference for the development of new therapeutic targets.

There are still some limitations in our study. First, the datasets we used to train and validate the two DL-models are not sufficiently large. Further incorporation of multicenter data to improve the generalization performance of the models will be an important consideration in the future before the models are widely used in clinical practice. Second, since our study is a retrospective one, our diagnostic and prognostic DL-models need to be further validated based on prospective, randomized, multicenter clinical trial formulated by SPIRIT-AI and CONSORT-AI [69], to improve clinicopathological information related to treatment and prognosis [7], including variant histology, adjuvant chemotherapy, type of surgery and comorbidities. Third, pathological slides obtained from different laboratories show variations due to differences among labs in sample collection, fabrication techniques, staining materials, and digital scans. Although we used a staining normalization approach and demonstrated decent performance for both models on an external validation set, challenges related to data normalization remain. Hence, it is necessary to establish a standardized procedure for the production of pathological slide images to improve the quality of the dataset.

## 5. Conclusions

We developed weakly supervised models for diagnosing BLCA and predicting OS based on DL using digital H&E-stained images of BLCA patients. The performance of the diagnostic model was comparable to that of expert uropathologists, and the prognostic model showed better prognostic value than any other clinical, biological, and pathological features. Our models can not only assist clinicians in recognizing BLCA, but also help stratify MIBC patients and improve subsequent personalized treatment decisions. Finally, further analysis of the critical tumor-related regions and MibcMLP-predicted risk scores increased the amount of knowledge garnered from WSIs and potentially led to new biomarker discoveries.

## Figures and Tables

**Table 1:** Clinical, biological, and pathological features of the MIBC patients included in the prognostic model (MibcMLP).

	TCGA (*N* = 326)	RHWU (*N* = 144)
Age (years)	68 (57, 79)	66 (26, 87)
Sex		
female	87 (26.69%)	21 (14.58%)
male	239 (73.31%)	123 (85.42%)
pT stage		
pT2	99 (30.37%)	58 (40.28%)
pT3	158 (48.47%)	67 (46.53%)
pT4	42 (12.88%)	19 (13.19%)
pTx	27 (8.28%)	0 (0%)
pN stage		
pN0	179 (54.91%)	71 (49.31%)
pN1	38 (11.66%)	37 (25.69%)
pN2	67 (20.55%)	20 (13.89%)
pN3	6 (1.84%)	16 (11.11%)
pNx	36 (11.04%)	0 (0%)
pM stage		
pM0	138 (42.33%)	140 (97.22%)
pM1	8 (2.45%)	4 (2.78%)
pMx	180 (55.22%)	0 (0%)
pTNM stage		
Stage II	106 (32.52%)	41 (28.47%)
Stage III	104 (31.90%)	81 (56.25%)
Stage IV	115 (35.28%)	22 (15.28%)
Missing	1 (0.31%)	0 (0%)
Lymphovascular invasion		
No	100 (30.67%)	91 (63.19%)
Yes	121 (37.12%)	53 (36.81%)
Missing	105 (32.21%)	0 (0%)
Survival status		
Alive	180 (55.21%)	79 (54.86%)
Dead	146 (44.79%)	65 (45.14%)
OS time (months)	18.1 (0, 165.6)	16.0 (1.9, 66.0)

MIBC, Muscle-invasive bladder cancer.

**Table 2:** Accuracy, sensitivity, specificity, and AUC of the diagnostic model (BlcaMIL) and human pathologists.

**a. Accuracy, Sensitivity, and Specificity in the Diagnostic Model (BlcaMIL)**					
		Accuracy (95% CI)	Sensitivity (95% CI)	Specificity (95% CI)	AUC (95% CI)
**Training set**		0.998 (0.996, 0.999)	0.999 (0.998, 1.000)	0.998 (0.996, 0.999)	1.000 (1.000, 1.000)
**Internal validation set**		0.998 (0.996, 1.000)	1.000 (1.000, 1.000)	0.996 (0.992, 1.000)	1.000 (1.000, 1.000)
**External validation set**		0.987 (0.981, 0.994)	0.984 (0.971, 0.998)	0.986 (0.979, 0.993)	0.993 (0.990, 0.997)
**b. Comparison of the BlcaMIL model and human pathologists in the external validation set**					
	Accuracy (95% CI)	Sensitivity (95% CI)	Specificity (95% CI)	*p*-Value *	Kappa ^#^
**BlcaMIL Model**	0.987 (0.981, 0.994)	0.984 (0.971, 0.998)	0.986 (0.979, 0.993)	-	-
**Expert Uropathologist A**	0.991 (0.987, 0.995)	0.988 (0.981, 0.995)	0.996 (0.989, 1.000)	1.000	0.909
**Expert Uropathologist B**	0.993 (0.991, 0.995)	0.991 (0.987, 0.995)	0.996 (0.989, 1.000)	1.000	0.925
**Junior Pathologist C**	0.876 (0.852, 0.900)	0.834 (0.811, 0.858)	0.940 (0.904, 0.976)	<0.0001	0.711

* A paired Chi-squared test (McNemar’s test) was used to examine differences in accuracy between the BlcaMIL model and each uropathologist. **^#^** Inter-observer agreement between the BlcaMIL model and each uropathologist assessed by the Cohen kappa coefficient. CI, Confidence Interval.

## Data Availability

The datasets of TCGA cohort for this study can be found in the [The Cancer Genome Atlas Program] [https://portal.gdc.cancer.gov/, accessed on 22 October 2022].

## Supplementary Materials

The following supporting information can be downloaded at: https://www.mdpi.com/article/10.3390/cancers14235807/s1, Figure S1. Heatmaps of diagnostic model (BlcaMIL) on WSI at different pathological stages in the RHWU cohort. A–C, pathological original images, corresponding heatmaps and representative patches with pathological TNM stage II(**A**), III (**B**), and IV(**C**) from the RHWU cohort, respectively. Figure S2. Prognostic value of MibcMLP-generated risk scores in training set. The p-value was evaluated by Log-Rank test; Table S1a. Dataset distribution of patients and corresponding images in the diagnostic model (BlcaMIL); Table S1b. Dataset distribution of patients and corresponding images in the prognostic model (MibcMLP); Table S1c. Dataset distribution of images in the training, internal validation and external validation sets. Table S2. Cox analyses of prognostic factors in the training set.
