# Supplementary material for: Accurate Diagnosis and Survival Prediction of Bladder Cancer Using Deep Learning on Histological Slides
Source: Cancers (Basel). 2022 Nov 25;14(23):5807. doi: 10.3390/cancers14235807 (PMC9737237; doi:10.3390/cancers14235807)
Supplement: Supplementary file 1 [file cancers-14-05807-s001.zip › cancers-2024115-supplementary.pdf]

cancers

MDPI

Supplementary Materials

Supplementary Table S1

(a) Dataset distribution of patients and corresponding images in the diagnostic model (BlcaMIL)				
	Patients	Total images	Malignant images	Normal images
TCGA	412	926	887 (95.79%)	39 (4.21%)/740 *
RHWU	250	250	150 (60%)	100 (40%)
(b) Dataset distribution of patients and corresponding images in the prognostic model (MibcMLP)				
	Patients	Images		
TCGA	326	326		
RHWU	144	144		
(c) Dataset distribution of images in the training, internal validation and external validation sets				
	Training set	Internal validation set	External validation set	
BlcaMIL	1302	325	250	
MibcMLP	190	136	144	

* The number of normal images from TCGA after data argumentation.

Supplementary Table S2. Cox analyses of prognostic factors in the training set.

9	Univariate analysis		Multivariate analysis	
	HR (95%CI)	*p*-value	HR (95%CI)	*p*-value
Age				
< 70	Ref.			
≥70	1.407 (0.928, 2.135)	0.108		
Sex				
female	Ref.			
male	1.003 (0.637, 1.579)	0.990		
pT stage				
pT1-2	Ref.		Ref.	
pT3-4	1.743 (1.083, 2.803)	0.022	1.347 (0.801, 2.267)	0.262
pN stage				
pN0-1	Ref.		Ref.	
pN2-3	2.118 (1.358, 3.303)	< 0.001	1.010 (0.535, 1.907)	0.975
pM stage				
pM0	Ref.		Ref.	
pM1	5.257 (2.258, 12.238)	< 0.001	1.418 (0.533, 3.773)	0.484
pTNM stage				
Stage I-II	Ref.		Ref.	
Stage III-IV	2.348 (1.537, 3.587)	< 0.001	1.705 (0.897, 3.240)	0.104
	Lymphovascular invasion			
No	Ref.		Ref.	
Yes	1.538 (1.013, 2.334)	0.043	1.020 (0.619, 1.680)	0.939
Risk score				
Low-risk score	Ref.		Ref.	
High-risk score	3.888 (2.811, 5.380)	< 0.001	3.474 (2.449, 4.929)	< 0.001

95% CI, 95% Confidence Interval.

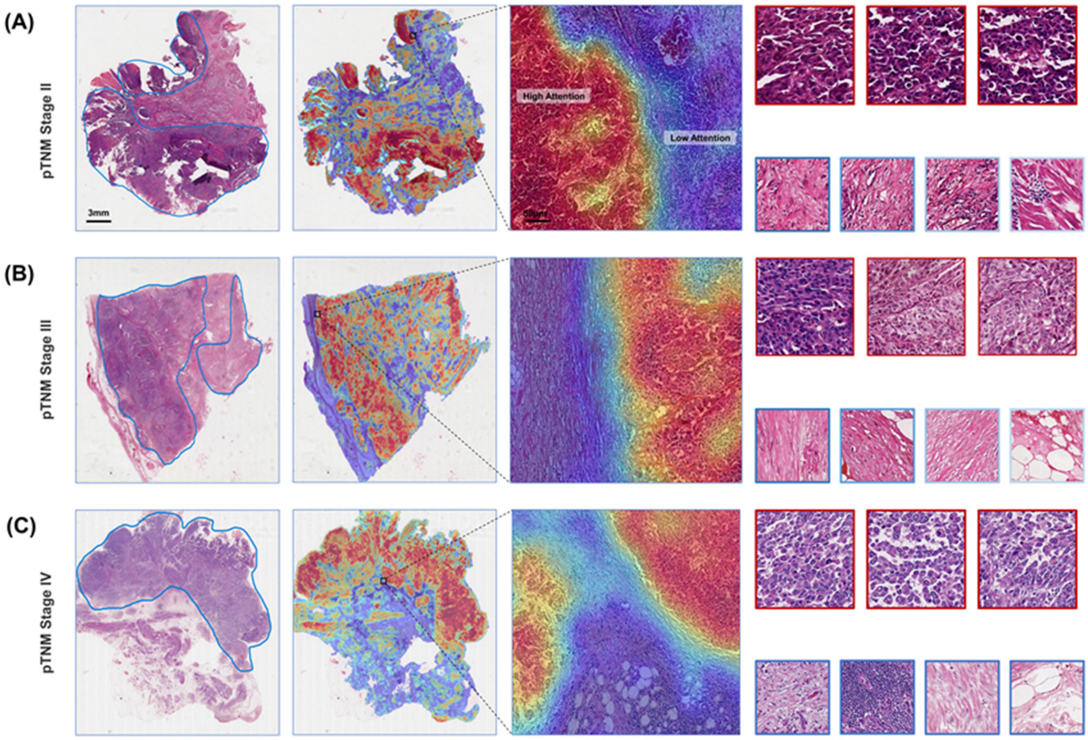

Supplementary Figure S1. Heatmaps of diagnostic model (BlcaMIL) on WSI at different pathological stages in the RHWU cohort. (**A–C**), pathological original images, corresponding heatmaps and representative patches with pathological TNM stage II, III, and IV from the RHWU cohort, respectively.

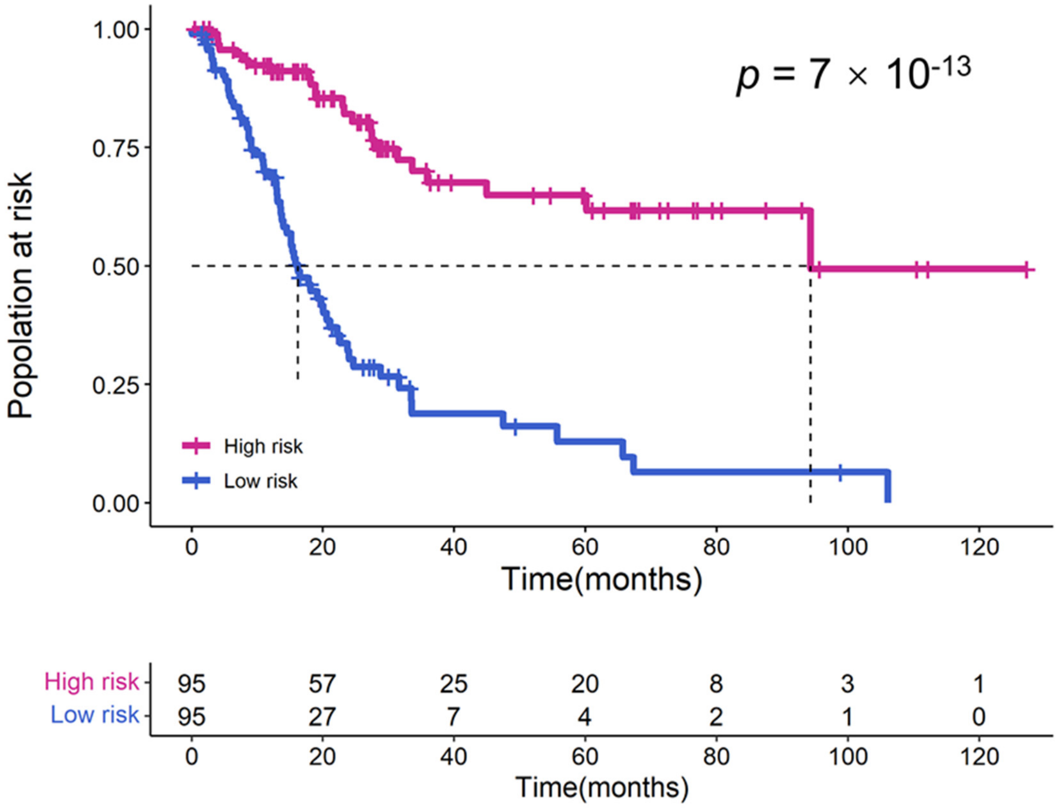

Supplementary Figure S2. Prognostic value of MibcMLP-generated risk scores in training set. The *p*-value was evaluated by Log-Rank test.
